# (*E*)-3-(4-Methyl­phen­yl)-1-(1,3-thia­zol-2-yl)prop-2-en-1-one

**DOI:** 10.1107/S1600536812019575

**Published:** 2012-05-26

**Authors:** Annamalai Palaniappan, Arumugam Arulmozhivarman, Rajamanickam Ramachandran, Subramanian Srinivasan, Sivakolunthu Senthan

**Affiliations:** a Department of Chemistry, Annamalai University, Annamalai Nagar 608 002, Tamil Nadu, India; b Department of Image Science and Engineering, Pukyong National University, Busan 608 737, Republic of Korea

## Abstract

In the title chalcone, C_13_H_11_NOS, derived from the condensation of *p*-tolualdehyde and 1-(1,3-thia­zol-2-yl)ethanone, the olefine group has a *trans* configuration. No classical hydrogen bonding is present in the crystal structure.

## Related literature

For background to thia­zoles, see: Fontecave *et al.* (2003 ▶); Kleemann *et al.* (2001 ▶) and for their biological activity, see: Bharti *et al.* (2010 ▶); Bell *et al.* (1995 ▶); Cortes *et al.* (2007 ▶).
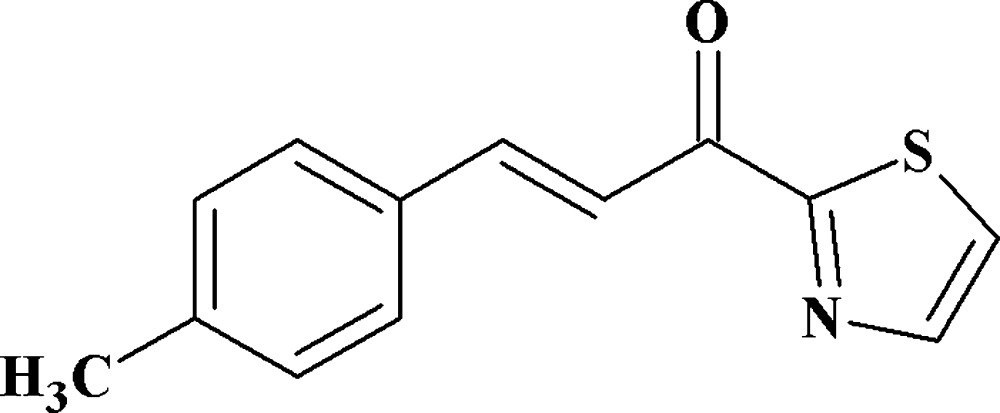


## Experimental

### 

#### Crystal data

C_13_H_11_NOS
*M* *_r_* = 229.29
Monoclinic, 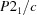

*a* = 13.9486 (9) Å
*b* = 11.1773 (8) Å
*c* = 7.4579 (5) Å
β = 102.061 (4)°
*V* = 1137.08 (13) Å^3^
*Z* = 4
Mo *K*α radiation
μ = 0.26 mm^−1^
*T* = 293 K
0.20 × 0.20 × 0.20 mm

#### Data collection

Bruker Kappa APEXII CCD diffractometer
Absorption correction: multi-scan (*SADABS*; Bruker, 1999 ▶) *T*_min_ = 0.949, *T*_max_ = 0.949
10320 measured reflections
2808 independent reflections
2070 reflections with *I* > 2σ(*I*)
*R*_int_ = 0.027

#### Refinement

*R*[*F* ^2^ > 2σ(*F* ^2^)] = 0.040
*wR*(*F* ^2^) = 0.118
*S* = 1.05
2808 reflections
153 parameters
H atoms treated by a mixture of independent and constrained refinement
Δρ_max_ = 0.23 e Å^−3^
Δρ_min_ = −0.24 e Å^−3^

### 

Data collection: *APEX2* (Bruker, 2004 ▶); cell refinement: *APEX2* and *SAINT-Plus* (Bruker, 2004 ▶); data reduction: *SAINT-Plus* and *XPREP* (Bruker, 2004 ▶); program(s) used to solve structure: *SIR92* (Altomare *et al.*, 1993 ▶); program(s) used to refine structure: *SHELXL97* (Sheldrick, 2008 ▶); molecular graphics: *ORTEP-3 for Windows* (Farrugia, 1997 ▶); software used to prepare material for publication: *WinGX* (Farrugia, 1999 ▶).

## Supplementary Material

Crystal structure: contains datablock(s) global, I. DOI: 10.1107/S1600536812019575/ez2285sup1.cif

Structure factors: contains datablock(s) I. DOI: 10.1107/S1600536812019575/ez2285Isup2.hkl

Supplementary material file. DOI: 10.1107/S1600536812019575/ez2285Isup3.cml

Additional supplementary materials:  crystallographic information; 3D view; checkCIF report

## Figures and Tables

**Fig. 1.**
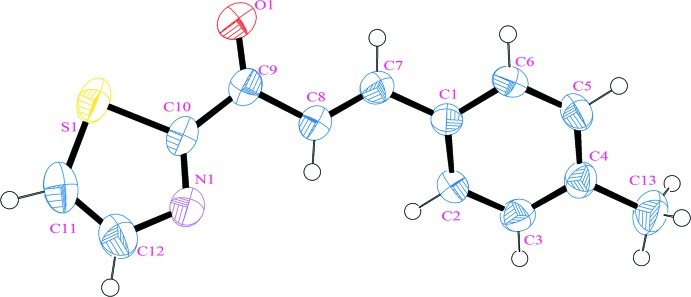
The ORTEP representation of title compound showing the atom numbering scheme and ellipsoids at the 50% probability level.

## supplementary crystallographic information

### Comment

Molecules which possess both sulphur and nitrogen atoms exhibit universal and crucial roles in living organisms (Fontecave *et al.*, 2003), with thiazoles and their derivatives being an important class of heterocyclic compounds (Kleemann *et al.*, 2001). Analogues of these are present in several drugs with a wide range of biological properties, such as antibacterial (Bharti *et al.*, 2010), antiviral (Bell *et al.*, 1995) and anticancer (Cortes *et al.*, 2007). Our research has been focused towards finding new therapeutic agents, using thiazole compounds. Similarly, several α,β-unsaturated ketones have been found to have good biological activity. Therefore, in this paper we report both the thiazole and α,β-unsaturated ketone moieties in one molecule. The title compound (Fig. 1) exists in an E configuration with respect to the C7-C8 double bond. Both phenyl and thiazole rings adopt planar orientations and there is no classical hydrogen bonding found.

### Experimental

To an aqueous ethanolic solution of p-tolualdehyde (0.01 mol) and 2-acetylthiazole (0.01 mol), a sodium hydroxide solution was added slowly and stirred until a precipitate formed. The obtained solid was filtered and washed well with water. Single crystals were grown by the slow evaporation technique using ethanol as solvent.

### Refinement

H-atoms were positioned and refined using a riding model, with aromatic C—H = 0.93 Å, methine C—H = 0.98 Å, methylene C—H = 0.97 Å and amino N—H = 0.83 and 0.94 Å. The displacement parameters were set for phenyl, methylene and aliphatic H atoms at *U*_iso_(H)=1.2*U*_eq_(C).

### Crystal data

**Table:**

C_13_H_11_NOS	*Z* = 4
*M**_r_* = 229.29	*F*(000) = 480
Monoclinic, *P*2_1_/*c*	*D*_x_ = 1.339 Mg m^−^^3^
Hall symbol: -P 2ybc	Mo *K*α radiation, λ = 0.71073 Å
*a* = 13.9486 (9) Å	µ = 0.26 mm^−^^1^
*b* = 11.1773 (8) Å	*T* = 293 K
*c* = 7.4579 (5) Å	Prism, colorless
β = 102.061 (4)°	0.20 × 0.20 × 0.20 mm
*V* = 1137.08 (13) Å^3^	

### Data collection

**Table:**

Bruker Kappa APEXII CCD diffractometer	2808 independent reflections
Radiation source: fine-focus sealed tube	2070 reflections with *I* > 2σ(*I*)
Graphite monochromator	*R*_int_ = 0.027
ω and φ scan	θ_max_ = 28.3°, θ_min_ = 1.5°
Absorption correction: multi-scan (*SADABS*; Bruker, 1999)	*h* = −18→17
*T*_min_ = 0.949, *T*_max_ = 0.949	*k* = −11→14
10320 measured reflections	*l* = −9→9

### Refinement

**Table:**

Refinement on *F*^2^	Primary atom site location: structure-invariant direct methods
Least-squares matrix: full	Secondary atom site location: difference Fourier map
*R*[*F*^2^ > 2σ(*F*^2^)] = 0.040	Hydrogen site location: inferred from neighbouring sites
*wR*(*F*^2^) = 0.118	H atoms treated by a mixture of independent and constrained refinement
*S* = 1.05	*w* = 1/[σ^2^(*F*_o_^2^) + (0.0598*P*)^2^ + 0.1832*P*] where *P* = (*F*_o_^2^ + 2*F*_c_^2^)/3
2808 reflections	(Δ/σ)_max_ = 0.001
153 parameters	Δρ_max_ = 0.23 e Å^−^^3^
0 restraints	Δρ_min_ = −0.24 e Å^−^^3^

### Special details

**Table:**

Geometry. All esds (except the esd in the dihedral angle between two l.s. planes) are estimated using the full covariance matrix. The cell esds are taken into account individually in the estimation of esds in distances, angles and torsion angles; correlations between esds in cell parameters are only used when they are defined by crystal symmetry. An approximate (isotropic) treatment of cell esds is used for estimating esds involving l.s. planes.
Refinement. Refinement of F^2^ against ALL reflections. The weighted R-factor wR and goodness of fit S are based on F^2^, conventional R-factors R are based on F, with F set to zero for negative F^2^. The threshold expression of F^2^ > 2sigma(F^2^) is used only for calculating R-factors(gt) etc. and is not relevant to the choice of reflections for refinement. R-factors based on F^2^ are statistically about twice as large as those based on F, and R- factors based on ALL data will be even larger.

### Fractional atomic coordinates and isotropic or equivalent isotropic displacement parameters (Å^2^)

**Table:**

	*x*	*y*	*z*	*U*_iso_*/*U*_eq_	
S1	0.98502 (3)	0.17057 (5)	1.16674 (6)	0.05963 (18)	
O1	0.82353 (10)	−0.00430 (12)	1.0300 (2)	0.0721 (4)	
N1	0.84607 (10)	0.31056 (13)	1.0198 (2)	0.0540 (4)	
C1	0.53219 (10)	0.08303 (13)	0.72422 (19)	0.0380 (3)	
C7	0.62986 (11)	0.05556 (15)	0.8309 (2)	0.0425 (3)	
C4	0.34124 (11)	0.13769 (15)	0.5336 (2)	0.0451 (4)	
C10	0.86817 (11)	0.19796 (15)	1.0453 (2)	0.0440 (4)	
C2	0.51393 (11)	0.18190 (13)	0.6090 (2)	0.0420 (3)	
H2	0.5657	0.2309	0.5948	0.050*	
C8	0.70403 (11)	0.13124 (16)	0.8719 (2)	0.0471 (4)	
C3	0.42049 (11)	0.20835 (15)	0.5155 (2)	0.0458 (4)	
H3	0.4102	0.2748	0.4388	0.055*	
C6	0.45308 (11)	0.01025 (14)	0.7382 (2)	0.0451 (4)	
H6	0.4635	−0.0578	0.8113	0.054*	
C9	0.79948 (11)	0.09785 (15)	0.9842 (2)	0.0476 (4)	
C5	0.35920 (12)	0.03767 (15)	0.6448 (2)	0.0497 (4)	
H5	0.3074	−0.0119	0.6570	0.060*	
C13	0.23967 (13)	0.16866 (19)	0.4303 (3)	0.0681 (5)	
H4C	0.2421	0.2405	0.3608	0.102*	
H4A	0.1977	0.1809	0.5156	0.102*	
H4B	0.2145	0.1043	0.3487	0.102*	
C12	0.92396 (14)	0.37879 (19)	1.1005 (3)	0.0646 (5)	
H12	0.9216	0.4619	1.0966	0.078*	
C11	1.00462 (14)	0.31959 (19)	1.1861 (3)	0.0622 (5)	
H11	1.0625	0.3554	1.2469	0.075*	
H7	0.6378 (13)	−0.0218 (18)	0.877 (2)	0.059 (5)*	
H8	0.6995 (14)	0.2095 (18)	0.835 (3)	0.066 (6)*	

### Atomic displacement parameters (Å^2^)

**Table:**

	*U*^11^	*U*^22^	*U*^33^	*U*^12^	*U*^13^	*U*^23^
S1	0.0395 (2)	0.0771 (4)	0.0562 (3)	0.0054 (2)	−0.00395 (18)	0.0080 (2)
O1	0.0545 (7)	0.0519 (8)	0.1003 (11)	0.0082 (6)	−0.0058 (7)	0.0108 (7)
N1	0.0422 (7)	0.0531 (9)	0.0628 (9)	0.0023 (6)	0.0019 (6)	−0.0033 (7)
C1	0.0372 (7)	0.0350 (7)	0.0417 (7)	0.0004 (6)	0.0080 (6)	−0.0042 (6)
C7	0.0412 (8)	0.0394 (8)	0.0465 (8)	0.0040 (6)	0.0079 (6)	−0.0002 (7)
C4	0.0383 (7)	0.0472 (9)	0.0476 (8)	0.0011 (6)	0.0040 (6)	−0.0089 (7)
C10	0.0333 (7)	0.0566 (9)	0.0405 (8)	0.0055 (7)	0.0041 (6)	0.0014 (7)
C2	0.0389 (7)	0.0395 (8)	0.0477 (8)	−0.0048 (6)	0.0096 (6)	0.0008 (6)
C8	0.0390 (8)	0.0444 (9)	0.0544 (9)	0.0022 (7)	0.0017 (7)	0.0025 (7)
C3	0.0469 (8)	0.0420 (8)	0.0465 (8)	0.0026 (7)	0.0046 (6)	0.0035 (7)
C6	0.0478 (8)	0.0341 (8)	0.0526 (9)	−0.0041 (6)	0.0083 (7)	0.0028 (7)
C9	0.0378 (7)	0.0517 (10)	0.0519 (9)	0.0049 (7)	0.0059 (6)	0.0019 (7)
C5	0.0410 (8)	0.0459 (9)	0.0619 (10)	−0.0110 (7)	0.0099 (7)	−0.0046 (7)
C13	0.0425 (9)	0.0759 (13)	0.0777 (13)	0.0042 (9)	−0.0063 (9)	−0.0058 (10)
C12	0.0560 (10)	0.0618 (12)	0.0722 (12)	−0.0096 (9)	0.0047 (9)	−0.0107 (10)
C11	0.0466 (9)	0.0835 (14)	0.0530 (10)	−0.0139 (9)	0.0025 (8)	−0.0082 (9)

### Geometric parameters (Å, º)

**Table:**

S1—C11	1.689 (2)	C2—C3	1.376 (2)
S1—C10	1.7185 (15)	C2—H2	0.9300
O1—C9	1.219 (2)	C8—C9	1.465 (2)
N1—C10	1.300 (2)	C8—H8	0.92 (2)
N1—C12	1.360 (2)	C3—H3	0.9300
C1—C2	1.390 (2)	C6—C5	1.383 (2)
C1—C6	1.392 (2)	C6—H6	0.9300
C1—C7	1.460 (2)	C5—H5	0.9300
C7—C8	1.322 (2)	C13—H4C	0.9600
C7—H7	0.929 (19)	C13—H4A	0.9600
C4—C5	1.383 (2)	C13—H4B	0.9600
C4—C3	1.388 (2)	C12—C11	1.346 (3)
C4—C13	1.505 (2)	C12—H12	0.9300
C10—C9	1.482 (2)	C11—H11	0.9300
			
C11—S1—C10	89.32 (9)	C4—C3—H3	119.4
C10—N1—C12	109.56 (16)	C5—C6—C1	120.99 (14)
C2—C1—C6	117.70 (13)	C5—C6—H6	119.5
C2—C1—C7	122.26 (13)	C1—C6—H6	119.5
C6—C1—C7	120.03 (14)	O1—C9—C8	124.27 (16)
C8—C7—C1	126.00 (15)	O1—C9—C10	119.91 (15)
C8—C7—H7	118.9 (11)	C8—C9—C10	115.81 (14)
C1—C7—H7	115.1 (11)	C6—C5—C4	120.98 (14)
C5—C4—C3	118.03 (14)	C6—C5—H5	119.5
C5—C4—C13	121.73 (16)	C4—C5—H5	119.5
C3—C4—C13	120.21 (16)	C4—C13—H4C	109.5
N1—C10—C9	124.58 (14)	C4—C13—H4A	109.5
N1—C10—S1	114.79 (12)	H4C—C13—H4A	109.5
C9—C10—S1	120.58 (12)	C4—C13—H4B	109.5
C3—C2—C1	121.05 (14)	H4C—C13—H4B	109.5
C3—C2—H2	119.5	H4A—C13—H4B	109.5
C1—C2—H2	119.5	C11—C12—N1	116.45 (19)
C7—C8—C9	122.88 (16)	C11—C12—H12	121.8
C7—C8—H8	122.7 (12)	N1—C12—H12	121.8
C9—C8—H8	114.4 (13)	C12—C11—S1	109.87 (15)
C2—C3—C4	121.20 (15)	C12—C11—H11	125.1
C2—C3—H3	119.4	S1—C11—H11	125.1
			
C2—C1—C7—C8	19.2 (2)	C7—C1—C6—C5	177.01 (15)
C6—C1—C7—C8	−159.68 (16)	C7—C8—C9—O1	9.7 (3)
C12—N1—C10—C9	176.99 (16)	C7—C8—C9—C10	−169.19 (15)
C12—N1—C10—S1	−0.58 (19)	N1—C10—C9—O1	−172.68 (16)
C11—S1—C10—N1	0.65 (14)	S1—C10—C9—O1	4.8 (2)
C11—S1—C10—C9	−177.02 (14)	N1—C10—C9—C8	6.3 (2)
C6—C1—C2—C3	1.5 (2)	S1—C10—C9—C8	−176.29 (12)
C7—C1—C2—C3	−177.40 (14)	C1—C6—C5—C4	0.6 (2)
C1—C7—C8—C9	178.32 (14)	C3—C4—C5—C6	1.3 (2)
C1—C2—C3—C4	0.3 (2)	C13—C4—C5—C6	179.72 (16)
C5—C4—C3—C2	−1.7 (2)	C10—N1—C12—C11	0.2 (2)
C13—C4—C3—C2	179.84 (16)	N1—C12—C11—S1	0.3 (2)
C2—C1—C6—C5	−2.0 (2)	C10—S1—C11—C12	−0.52 (15)
